# The long-term impact of community mobilisation through participatory women's groups on women's agency in the household: A follow-up study to the Makwanpur trial

**DOI:** 10.1371/journal.pone.0197426

**Published:** 2018-05-14

**Authors:** Lu Gram, Jolene Skordis-Worrall, Dharma S. Manandhar, Daniel Strachan, Joanna Morrison, Naomi Saville, David Osrin, Kirti M. Tumbahangphe, Anthony Costello, Michelle Heys

**Affiliations:** 1 Institute for Global Health, University College London, London, United Kingdom; 2 Mother and Infant Research Activities (MIRA), Kathmandu, Nepal; 3 Department of Maternal, Newborn, Child and Adolescent Health (MCA), World Health Organization, Geneva, Switzerland; 4 Great Ormond Street Institute of Child Health, University College London, London, United Kingdom; Emory University, School of Public Health, UNITED STATES

## Abstract

Women’s groups practicing participatory learning and action (PLA) in rural areas have been shown to improve maternal and newborn survival in low-income countries, but the pathways from intervention to impact remain unclear. We assessed the long-term impact of a PLA intervention in rural Nepal on women’s agency in the household. In 2014, we conducted a follow-up study to a cluster randomised controlled trial on the impact of PLA women’s groups from 2001–2003. Agency was measured using the Relative Autonomy Index (RAI) and its subdomains. Multi-level regression analyses were performed adjusting for baseline socio-demographic characteristics. We additionally adjusted for potential exposure to subsequent PLA groups based on women’s pregnancy status and conduct of PLA groups in areas of residence. Sensitivity analyses were performed using two alternative measures of agency. We analysed outcomes for 4030 mothers (66% of the cohort) who survived and were recruited to follow-up at mean age 39.6 years. Across a wide range of model specifications, we found no association between exposure to the original PLA intervention with women’s agency in the household approximately 11.5 years later. Subsequent exposure to PLA groups was not associated with greater agency in the household at follow-up, but some specifications found evidence for reduced agency. Household agency may be a prerequisite for actualising the benefits of PLA groups rather than a consequence.

## Introduction

Women’s groups practising participatory learning and action (PLA) in rural areas have been shown to improve maternal and newborn survival in low-income countries [1]. Inspired by the philosophy of “popular education” by Paulo Freire [2], PLA interventions employ a trained facilitator to hold regular community meetings in which groups of local women are led through a cycle of problem identification, prioritisation, action planning, strategy implementation and outcome evaluation. The implementation and scale-up of PLA strategies to improve newborn mortality and health is explicitly endorsed in a recommendation by the World Health Organisation [3].

Despite strong evidence to support efficacy, the pathways from intervention to impact remain unclear and the evidence on how community-level exposure to PLA women’s groups achieves its impact is evolving [4,5]. Victora [5] commented on the long causal chain from ‘critical consciousness to mortality’ and compared the relative lack of theory on how PLA women’s groups currently work to the situation of 19th century epidemiologists before the discovery of germ theory—These had clear evidence for an association between poor sanitation and incidence of infectious disease, but a poor understanding of the biological mechanism.

Intermediary effects have been explored through qualitative studies, which have reported that women and men participating in the groups engaged in several collective action strategies from setting up mother and child health funds to producing and selling their own clean delivery kits. Women’s group members ascribed to attendance increased self-confidence and self-esteem and improved social support from the community [6–11]. However, almost no qualitative or quantitative evidence exists on the impact of PLA groups on women’s agency in the household.

Yet, agency in the household may be a key barrier to improved maternal, child and reproductive health. The United Nations Global Strategy for Women's, Children's and Adolescents' Health 2016–2030 recognises that “women, children and adolescents are potentially the most powerful agents for improving their own health and achieving prosperous and sustainable societies” [12]. At the same time, the 5th Sustainable Development Goal explicitly sets out to achieve gender equality and empower women and girls by 2030 [13].

Although no consensus currently exists regarding a single definition of empowerment and agency [14,15], a considerable body of literature discusses the range of possible meanings [14,16–21]. We follow Ibrahim and Alkire [19] in defining empowerment as “the expansion of agency” and Sen [22] in defining a person’s agency as “what the person is free to do and achieve in pursuit of whatever goals or values he or she regards as important” (p.203). Agency relates here to the extent to which individuals are able to provide reasons for their actions that accord with their own goals or values [22,23]. “Household agency” refers to individuals’ ability to pursue and achieve valued goals within the context of their personal relationships with members of their own household.

PLA groups might enable women to tackle structural obstacles to health such as low status in their own family, or household restrictions on the freedom of women to move and speak as they please [24,25]. In turn, lack of household agency has been linked to poorer fertility [26], child nutrition [27], health-seeking [28], an maternal and child health [28] outcomes in multiple contexts. One before-and-after analysis of a PLA intervention in rural Bangladesh found evidence for increased participation in household decision-making on women’s own healthcare [29]. Another evaluation of a cluster-randomised controlled trial of a PLA intervention to reduce low birth weight in Nepal found no evidence for an impact on household agency across a wide range of different indicators [30].

We followed up a closed cohort of surviving mother-infant dyads from the first trial of PLA women’s groups in Makwanpur District, Nepal from 2001–2003 [31]. Using data collected from this cohort, on average 11.5 years after the original trial, we sought to answer the following research question:

- What is the long-term impact of women’s residence in a community exposed to PLA groups during the original Makwanpur trial from 2001–2003 on women’s household agency at follow-up in 2014?

## Methods

### National context

Nepal remains one of the poorest countries in the world with 25.2% of the population living below the national poverty line [32]. However, the country has recently seen rapid economic growth with GNI per capita increasing from USD 230 in 2,000 to USD 730 in 2016 [32]. In particular, remittance income from foreign labour migration has increased dramatically from 2% in 2000 to 32% of GDP in 2017 [33]. Locally, agriculture constitutes 73% of total national employment [34]. Access to technology has improved markedly. In 2006, 51% of married households accessed electricity, 13% had a television, 6% a mobile phone, 4% a refrigerator and 4% a motorcycle [35]. In 2016, 91% accessed electricity, 52% had a television, 93% a mobile phone, 16% a refrigerator and 19% a motorcycle [36].

Between 1997–2007, Nepal experienced a violent civil war between Maoist rebels and government forces that ended in a peace agreement where it was decided that Nepal would transition from a parliamentary monarchy to a federal republic. After the election of a Constituent Assembly in 2008, a protracted period of negotiation followed ending in the adoption of a new constitution for Nepal in 2015. National and local elections were held under the new constitution in 2017. As local elections had been suspended in Nepal since 1997, this constituted the first set of local elections in Nepal in 20 years.

A major ideological platform for the Maoist party was the elimination of class, caste, ethnic, and gender inequality. Substantial numbers of women were recruited into its army during the civil war, while its women’s wing campaigned on issues of domestic violence and unequal inheritance rights [37,38]. In 2007, the Maoist party successfully pushed for a legal provision reserving 33% of Constituent Assembly seats for women, which was carried over into the electoral rules under the new constitution in 2015 [37]. However, the Maoist party has also been heavily criticised for pursuing elite politics to the neglect of women’s interests at the grassroots level and leaving a considerable gap between its rhetoric and its own practices [37–39].

In terms of quantitative indicators of women’s position, female literacy rates among ages 15–24 moderately increased from 2000 (60%) to 2015 (80%) [40], while female labour force participation remained approximately constant (81% in 2000, 83% in 2017) [41]. At the same time, the number of married women reporting making household decisions jointly or alone increased between 2001 (26% own health care, 30% large household purchases, 36% visits to family and friends) and 2006 (47% own health care, 53% major purchases, 57% visits to family and friends) [35,42]. However, between 2006 and 2016, little change was observed in these indicators (58% participated in decisions on own health care, 53% major purchases, 56% visits to family and friends in 2016) [36].

### Trial setting

Makwanpur District is a rural hill area in the central region of Nepal with a population that was approximately 400,000 in 2011 [43]. The district is divided into Village Development Committees (VDCs), which are further sub-divided into wards. Each VDC has an average population of 9500 and most households depend on subsistence agriculture. The majority ethnic group are Tibeto-Burman Tamangs, one of a wider group of traditionally non-Hindu ethnicities in Nepal called “Janajatis”. A distinguishing feature of Tamang gender norms is the relative lack of emphasis on control over female sexual purity and physical mobility as compared to Hindu gender norms [44].

### Original cRCT of PLA women’s groups

The UCL Institute for Global Health, in partnership with Nepalese NGO Mother and Infant Research Activities (MIRA), conducted the cluster RCT in 2001–2003 [31]. The intervention consisted of community-based PLA women’s groups facilitated by a local lay facilitator conducting monthly group meetings, in which members explored issues around pregnancy, childbirth and newborn health. Inclusion criteria for enrolment in the original trial evaluation required women to be pregnant, married and aged between 15 and 49, while women who were unmarried, permanently separated or widowed were excluded. The unit of randomisation was the VDC. 24 of the 43 VDCs within Makwanpur were selected randomly and pair-matched on topographic stratification, ethnic group composition and population density. One cluster of each pair was allocated randomly to the intervention arm. At the end of the trial, a 30% reduction in neonatal mortality and a 78% reduction in maternal mortality was observed in deliveries occurring in intervention compared to control clusters [31].

### Subsequent implementation of PLA groups

**Fig 1:**
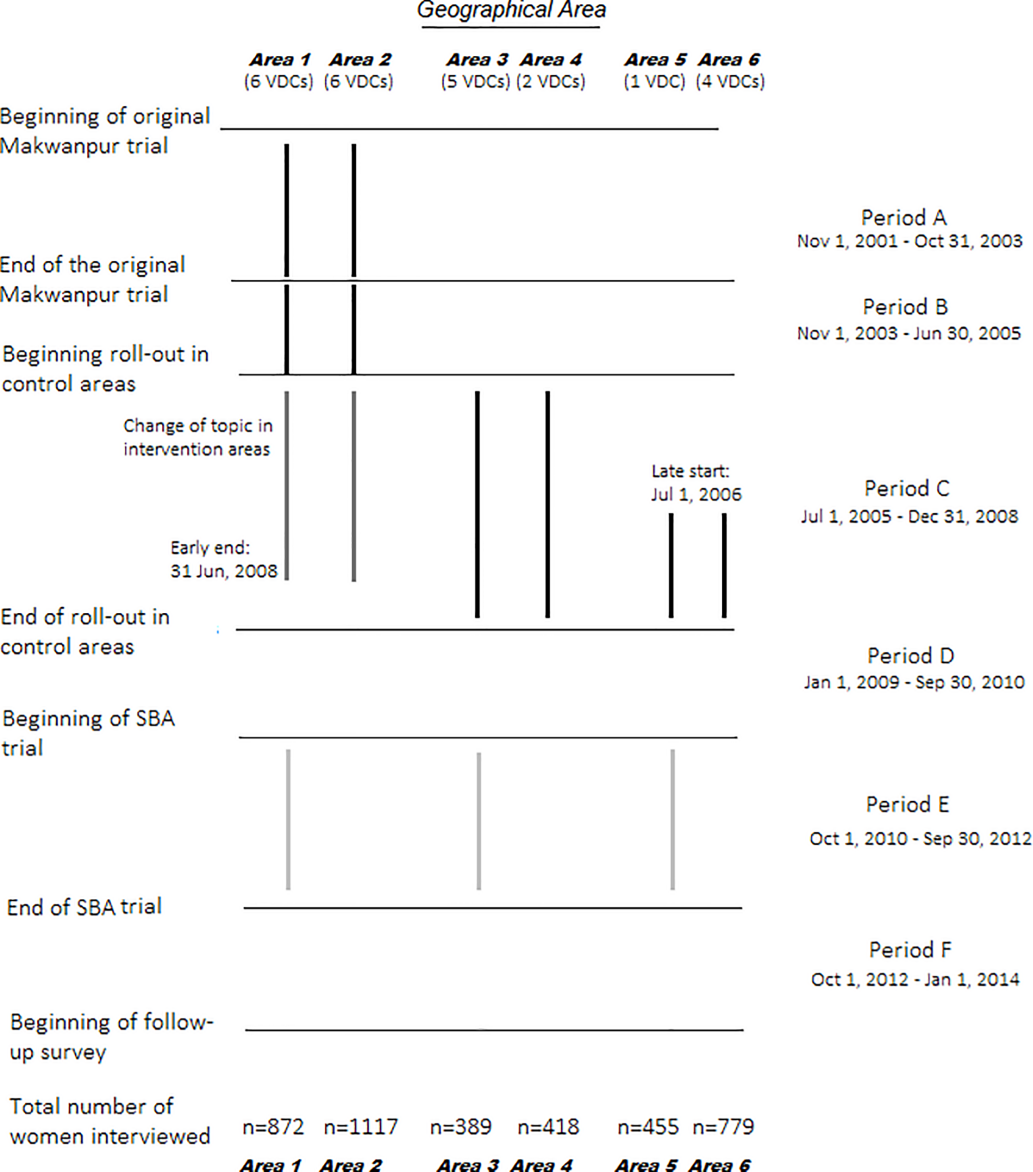
Implementation of participatory women’s groups. Vertical lines indicate exposure to an intervention. The total number of women interviewed refers to the number of women for which their household agency was measured at endline.

Following the original trial completion, there were additional periods in which MIRA implemented PLA groups. A representation of time periods and their attendant interventions are provided in Fig 1.

In the immediate post-trial period, due to the large observed impact on mortality, UCL and MIRA implemented the original intervention in control areas. Meanwhile, a revised PLA group intervention focusing on care-seeking for childhood illness and additionally involving men in maternal and newborn health was rolled out in the intervention arm. These groups continued until January 2009 when all group activities were suspended in preparation for a new trial, the “Skilled Birth Attendant Trial” (SBA trial). The SBA trial combined PLA groups with strengthening of Health Management Committees to increase skilled birth attendance [45]. All of the 43 VDCs in Makwanpur district were randomised to intervention or control (independent of previous randomisation in the original trial), with 21 in the intervention arm and 22 in the control. No PLA groups were run in control clusters of the SBA trial by UCL or MIRA. The trial ran from 2010 to 2012, after which all activities closed. The curricula of the three different models of PLA group meetings are outlined in S1 Table. Throughout the period from 2001 until 2012, newborn surveillance continued to identify newly delivered women.

### Follow-up study data collection

Our follow-up study cohort consisted of women who were enrolled in the original Makwanpur trial and had therefore given birth at least once between Nov 1, 2001 and Oct 31, 2003. In January 2014, field workers established contact with maternal-infant dyads from the closed cohort. Face-to-face interviews were conducted with contactable and willing participants by pairs of trained field interviewers and assistants [31]. Data collection was conducted in two waves in order to reduce the impact of the length of the interviews. Wave 1 was conducted between Jan and July 2014. Wave 2 was conducted between Sept and Dec 2014. All data were collected on android tablets using CommCare HQ [46,47]. To train field workers before administering the main survey and revise the final questionnaires based on interviewer feedback, pilot data were collected in both waves before field workers collected data for analysis. The first wave of data collection was piloted with 531 randomly sampled mother-child pairs, while the second was piloted with 141 mother-child pairs. Pilot data were excluded from this analysis.

### Analytic strategy

#### Choice of outcome measure

Our primary measure of agency in the household was the Relative Autonomy Index (RAI) [19,48]. Alkire [49,50] has repeatedly emphasised that the autonomy construct measured by the RAI closely approximates Sen’s [22] notion of agency. The RAI measures the extent to which individual behaviour is guided by internal relative to external drivers of action [51]. The RAI has been locally adapted and validated for use in Nepal [36], Bangladesh [52] and Chad [53] and suggested for use as an internationally comparable measure of empowerment [19]. The RAI has been used in impact evaluations of community-based health interventions in India [54] and Nepal [30] and regularly features in impact evaluations of health promotion interventions in high-income settings [55]. Elements of the RAI also form part of the Women’s Empowerment in Agriculture Index (WEAI) [56], which has been used in studies of maternal and child nutrition in Bangladesh [57], Ghana [58] and Nepal [59,60].

Decision-making questions are widely used in impact evaluations of women’s household agency [17,61,62], but do not map exactly onto our notion of ‘agency’ as conceptualised in this article [48,63]. This is because decision-making questions measure ‘the powers that they have even if they do not value these’ (p. 5) rather than ‘the empowerment that people value’ (p. 5) [64]. For the same reason, we also chose not to use the WEAI to directly measure agency, as it relies heavily on decision-making questions. However, we did choose to use a question on women’s ‘financial power’ or their perceived ability to participate in decisions on major expenditures in the household as a sensitivity measure due to its widespread use in impact evaluation studies (e.g. [62]).

Global questions on perceived overall influence in one’s life, such as the Power Ladder [65], do implicitly measure the powers that people value, as they do not pre-specify the types of powers that individuals should be able to affect. Nevertheless, such questions also differ from our conceptualisation of agency by focusing on opportunities for choice rather than experienced reasons for enacted actions [66]. While the availability of choice and the experience of internal motivation are likely positively correlated, they are not necessarily so, and may even conflict with one another [67]. As such, we chose to use the Power Ladder question as a sensitivity measure due to its open-ended, domain-independent nature.

#### Scoring the Relative Autonomy Index

The RAI measures household agency in four domains: (1) work outside the household, (2) domestic work, (3) health-seeking and (4) group participation. After a framing question on the activities, individuals carry out in each domain, respondents are asked if they perform these activities for internal reasons (e.g. because they want to or feel the activities are personally important) or for external reasons (e.g. because they fear angering family members otherwise). S2 Table shows the full list of items along with their scoring. The overall agency freedom score has a maximum of +12 and a minimum of -12. The 4 sub-domains (work outside the household, domestic work, health-seeking and group participation) are similarly scored and have a maximum of +3 and a minimum of -3.

#### Exposure

Our primary exposure variable was residence in an intervention (*Areas 1* and *2* in Fig 1) versus control (*Areas* 3 to 6) area in the original trial.

#### Measures of subsequent potential exposure to PLA groups

Subsequent potential exposure to PLA groups in the years following trial completion was measured in three ways to describe and test model assumptions around potential community- and individual-level exposure. First, we calculated a woman’s total years of residence in an area exposed to PLA interventions across Periods B to F (denoted by variable **RESEXP**, recorded in years). Second, using ongoing newborn surveillance data from 2003 to 2012, we calculated years of residence in an area running PLA groups while pregnant (denoted by **PREGEXP**, recorded in years). Since women could be pregnant multiple times from 2003 to 2012, this duration could in theory be arbitrarily large. Our assumption was that all pregnancies were detected by the surveillance system. Third, to model our assumptions around our measure of pregnancy-associated exposure time, we created a third variable **IMPEXP(θ)** (in units of years of exposure):

IMPEXP(θ)=(1−θ)PREGEXP+θRESEXP

**IMPEXP** imputes an exposure level for women based on a linear combination of the **PREGEXP** and the **RESEXP** variables. When **θ** is close to 0, we rely exclusively on pregnancy-associated exposure time. As **θ** approaches 1, we increasingly give weight to non-pregnancy associated exposure as well.

#### Potential confounders

Maternal education and age (years), household asset score index, caste/ethnicity, household occupation and sex of household head are both potentially important determinants of women’s agency in the household and potential predictors of loss to follow-up. The effect of **IMPEXP(θ)** on women’s agency is confounded by unobserved characteristics associated with women’s ability to make household decisions on their own fertility. To proxy for baseline ability to control one’s own fertility, we additionally controlled for the estimated total number of deliveries women had had upon enrolment into the original trial (called **DELIVERIES**). We estimated this number by subtracting the number of deliveries detected by pregnancy surveillance after the original trial had ended (Periods B-F in Fig 1) by the total number of deliveries reported at endline.

#### Statistical methods

First, we described differences in baseline characteristics during the original trial between intervention and control arms among women with complete data at follow-up and women with incomplete data. A complete-case analysis was conducted in case of missing data. Second, we compared crude averages of agency scores between women who had been resident in intervention and control clusters. Third, three-level hierarchical linear regression analyses were conducted with random intercepts for clusters based on VDCs and cluster pairings from the pair-matching process in the original trial with overall agency freedom as the main outcome. The four sub-domains of agency freedom (agency in work outside the household, domestic work, health-seeking, and group participation) were similarly analysed as secondary outcomes. For all 5 outcomes, effect sizes in standard deviations were reported.

We compared the results of the following six models with each of these 5 outcome measures. Models 1 to 5 use the entire study cohort. Model 6 is restricted to women who were not mothers-in-law at follow-up and who never delivered in periods subsequent to the original trial (Periods B-F in Fig 1).

- Model 1: unadjusted association of residence in intervention or control arm of the original trial with household agency.
- Model 2: Model 1, additionally adjusted for maternal education and age, household asset score index, caste/ethnicity, household occupation and sex of household head, all measured at baseline.
- Model 3: Model 2, additionally adjusted for **IMPEXP(0)** and **DELIVERIES.**
- Model 4: Model 2, additionally adjusted for **IMPEXP(0.5)** and **DELIVERIES**.
- Model 5: Model 2, additionally adjusted for **IMPEXP(1)** and **DELIVERIES**.
- Model 6: Model 5, using the restricted sample.

Model 1 is our primary model. Model 2 considers the impact of trial arm imbalances or missing data. Models 3–6 correct for exposure after the end of the original trial. Model 6 restricts the sample to the women who were least likely to have taken an interest in the women’s groups after the end of the original trial. In Models 3 to 6 we additionally checked for multicollinearity in the use of **IMPEXP(θ)** as a control variable by calculating Variance Inflation Factors and assessing the effect of adjusting for **IMPEXP(θ)** on the width of confidence intervals around our main impact estimate. A Variance Inflation Factor below 10 is traditionally considered low [68]. All models from 2 to 6 include covariates adjusting for baseline covariates. We found no difference between entering the covariates into our models directly or using a propensity score created from those covariates. In all reported analyses in the results section, the covariates were directly entered into the regression model.

Impacts on financial power and the Power Ladder question were analysed using the same model specifications 1–6. For financial power we used logistic regression model with fixed cluster pairing effects and Huber robust adjustment for clustering (effect sizes in logits). For the Power Ladder question, we used a hierarchical linear model with clustering at the level of the VDC and VDC pairing (effect sizes in raw steps on the ladder).

All statistical analyses were carried out in Stata 13 MP [69].

## Results

### Trial profile and descriptive statistics

**Fig 2:**
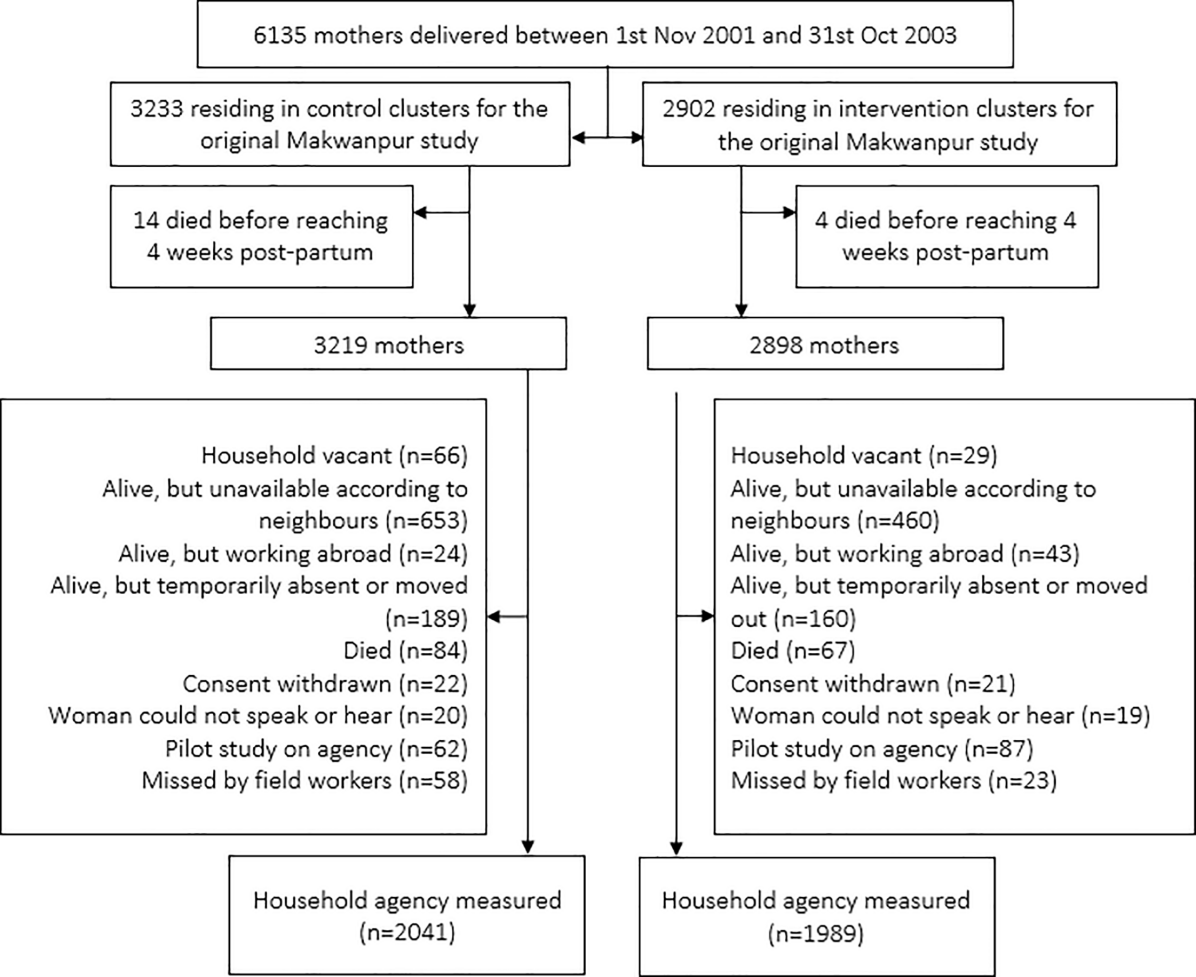
Trial flow for the data collection process.

Fig 2 displays the trial profile. In the original Makwanpur trial, 2902 women were sampled from intervention areas and 3233 from control areas. A total of 6117 mothers survived to 4 weeks postpartum. Excluding pilot surveys, 4030 mothers (68%) were available for interview, 2041 in control (65%) and 1989 in intervention (71%) VDCs. The main reasons for loss to follow-up were temporary absence or women having moved out of the study area (n = 1624) and death (n = 151). The mean age of women at follow-up was 39.6 years. No evidence for a systematic difference in follow-up rates was found (p = 0.31).

Table 1 compares baseline characteristics of women between intervention and control groups. The population of women were primarily of Janajati ethnicity (77%) with a household economy that revolved around agricultural labour (91%). Most women had no education (81%) and lived with male household heads (93%). We did not find any meaningful imbalances between intervention and control on baseline characteristics among women who were available for interview at follow-up, although the proportion of Brahmin/Chhetri caste women in the intervention group (17%) was slightly higher than control (12%). Of the original 6135 women who delivered during Period A, 12%, 31%, 2% and 2% delivered again and were detected by newborn surveillance in Periods B, C, D and E respectively (Fig 1).

**Table 1:** Baseline characteristics disaggregated by availability for interview. All values except deliveries in Periods B-E (see Fig 1) refer to measurements at baseline.

	Status at follow-up in 2014				
	*Available for interview*		*Lost to follow-up*		
	Exposure to the original Makwanpur trial		Exposure to the original Makwanpur trial		
	*Intervention*	*Control*	*Intervention*	*Control*	Total count
Total no. of women	1989	2041	826	1129	5985
**Caste**					
Janajati	75% (1495)	82% (1670)	67% (548)	80% (899)	77% (4612)
Newar	3% (50)	2% (38)	3% (23)	2% (23)	2% (134)
Brahmin/Chhetri	17% (344)	12% (239)	24% (197)	13% (142)	15% (922)
Dalit	4% (83)	4% (73)	5% (44)	5% (52)	4% (252)
Other	1% (12)	1% (19)	1% (6)	1% (7)	1% (44)
**Primary household occupation**					
Agriculture	88% (1743)	95% (1945)	85% (696)	95% (1071)	91% (5455)
Waged labour	8% (154)	3% (63)	10% (79)	2% (24)	5% (320)
Salaried/government job	3% (54)	1% (17)	3% (26)	1% (11)	2% (108)
Small business	2% (33)	1% (14)	2% (17)	2% (17)	1% (81)
**Asset score**					
None	52% (1027)	56% (1139)	51% (416)	56% (634)	54% (3216)
Clock, radio, iron or bicycle	32% (630)	35% (722)	31% (256)	35% (388)	33% (1996)
More costly assets	16% (327)	9% (178)	18% (146)	9% (101)	13% (752)
**Maternal education**					
No education	77% (1531)	87% (1768)	70% (575)	85% (956)	81% (4830)
1–3 years	8% (159)	4% (87)	8% (63)	5% (53)	6% (362)
4–8 years	12% (247)	8% (153)	18% (149)	8% (93)	11% (642)
9+ years	2% (47)	2% (31)	4% (31)	2% (21)	2% (130)
**Maternal age**					
<25 years' old	42% (840)	39% (789)	50% (413)	46% (512)	43% (2554)
25–29 years' old	26% (520)	27% (545)	25% (206)	22% (248)	25% (1519)
30+ years' old	31% (624)	35% (705)	24% (199)	32% (363)	32% (1891)
**Gender of household head**					
Male	92% (1819)	95% (1936)	90% (737)	96% (1077)	93% (5569)
Female	8% (165)	5% (103)	10% (81)	4% (46)	7% (395)
**Deliveries**					
Delivered in Period A	100% (1989)	100% (2041)	100% (826)	100% (1129)	100% (5985)
Delivered in Period B	15% (290)	11% (233)	12% (103)	9% (107)	12% (733)
Delivered in Period C	29% (572)	41% (845)	20% (164)	27% (301)	31% (1882)
Delivered in Period D	1% (26)	3% (56)	1% (9)	1% (13)	2% (104)
Delivered in Period E	2% (42)	4% (73)	1% (7)	2% (17)	2% (139)
**Missing values (n)**	5	2	8	6	21

To check for bias due to missing data, we compared women who were interviewed with women who were lost to follow-up. The distribution of caste, household occupation, assets, education, maternal age and gender of the household head among respondents with missing data closely tracked the distribution of corresponding variables among women who were interviewed. Tests of interaction between availability for interview at endline and intervention/control status revealed no evidence for differential loss to follow-up with respect to caste group, asset score, maternal age, education or baseline number of deliveries by trial allocation (*p*-values all >0.05).

Table 2 displays characteristics of women collected at follow-up, which provide key context for our findings. Nearly all women worked outside the home (99%), while about half were the main person responsible for cooking, cleaning and laundry in the house (54%), and about a third were responsible for money management and shopping for the household (36%). Most women sought health care in a facility for a moderately severe illness (64%), while the rest primarily reported going to the local pharmacy or shop (33%). 66% of women participated in groups, but the overwhelming majority of these considered financial groups the main type of group that they currently participated in—these groups are primarily Rotating Savings and Credit Associations (ROSCAs) or Accumulating Savings and Credit Associations (ASCAs). Only 2% of women considered a health group as their main group. 6% of women participated in any health group (not shown).

**Table 2:** Descriptive statistics collected from respondents upon follow-up. n = 4030. †6 respondents had missing values on health-seeking behaviour. ‡19 respondents had missing values on group participation. • Values reported in the format mean (SD). Scores for agency freedom are raw, unstandardized scores.

		Exposure to the original Makwanpur trial		
		*Intervention*	*Control*	*Overall*
Sample size		1989	2041	4030
**Descriptive characteristics**				
Employment	Respondent works outside the home	99% (1963)	100% (2032)	99% (3995)
	Respondent does not work outside	1% (26)	0% (9)	1% (35)
Domestic work	Respondent is main person responsible for cooking, cleaning and laundry	46% (924)	61% (1237)	54% (2161)
	Respondent is responsible for managing money and doing the shopping	41% (825)	30% (615)	36% (1440)
Place of health-seeking†	Public/private facility	75% (1493)	52% (1064)	64% (2557)
	Pharmacy/shop	22% (444)	43% (882)	33% (1326)
	Other (incl. traditional healer)	1% (10)	2% (45)	1% (55)
	Doesn't do anything	2% (42)	2% (44)	2% (86)
Group participation‡	*Does not participate*	26% (515)	43% (866)	34% (1381)
	*Participates*			
	Mainly employment-related	2% (38)	3% (69)	3% (107)
	Mainly finance-related	68% (1344)	49% (995)	58% (2339)
	Mainly health-related	2% (35)	2% (32)	2% (67)
	Other	3% (56)	3% (61)	3% (117)
**Outcome measures**				
Agency freedom•	Overall agency (-12 to +12)	9.9 (2.0)	9.4 (2.3)	9.6 (2.2)
	Agency in employment (-3 to +3)	2.6 (0.7)	2.8 (0.6)	2.7 (0.7)
	Agency in domestic work (-3 to +3)	2.8 (0.6)	2.8 (0.5)	2.8 (0.6)
	Agency in health-seeking (-3 to +3)	2.2 (0.8)	2.0 (0.8)	2.1 (0.8)
	Agency in group participation (-3 to +3)	2.2 (1.1)	1.8 (1.3)	2.0 (1.2)
Financial decision-making power	Only woman herself makes decisions	11% (222)	10% (195)	10% (417)
	Woman and other household members make decisions jointly	77% (1531)	80% (1633)	79% (3164)
	Woman not involved in decisions	12% (236)	10% (213)	11% (449)
Ladder question	Current step (1 to 10)	4.2 (1.5)	4.6 (1.6)	4.4 (1.6)

Table 2 also displays descriptive statistics for our main outcome measure in raw, unstandardised units. On measures of agency freedom, most women scored in the high range. The mean overall agency freedom score for all women, irrespective of trial arm, was 9.6 (standard deviation 2.2). 75% of women achieved the maximum score in the employment domain and 82% achieved the maximum score in the domestic work domain, while 37% and 47% achieved the maximum in health-seeking and group participation domains, respectively (not shown in table). 19% of women achieved the maximum score on overall agency freedom. Similarly, 89% of women reported being involved in decisions on major expenditures in the household, with 70% of women reporting making decisions jointly with their husband. However, in the Power Ladder question, mean scores were much lower (4.4 with SD 1.6), as 82% of women placed themselves on Step 5 or lower out of 10.

### Intervention impacts on household agency as measured by the RAI

Table 3 shows impact estimates for the original Makwanpur intervention under different model specifications on overall household agency. There was a non-statistically significant positive relation between being resident in an intervention cluster at the time of the original cRCT and overall agency at follow-up (*p*-values all >0.21).

**Table 3:** Impact estimates of the effect of exposure to the original Makwanpur intervention on overall agency measured with the RAI. Effect sizes in standard deviations. 95% confidence intervals in brackets.

*Outcome*: *Overall agency freedom*						
Model no.	1	2	3	4	5	6
*Exposure*: *Original exposure to PLA groups at cRCT*						
Original trial intervention	0.18 (-0.31, 0.68)	0.17 (-0.32, 0.66)	0.17 (-0.32, 0.65)	0.26 (-0.24, 0.77)	0.08 (-0.51, 0.66)	0.12 (-0.47, 0.71)
*Exposure*: *Subsequent exposure to PLA groups*						
IMPEXP(0)			-0.07* (-0.14, -0.01)			
IMPEXP(0.5)				-0.13*(-0.25, -0.01)		
IMPEXP(1)					0.05 (-0.16, 0.26)	0.01 (-0.20, 0.22)
DELIVERIES^#^			-0.03*** (-0.04, -0.01)	-0.03*** (-0.04, -0.01)	-0.03*** (-0.04, -0.01)	-0.04*** (-0.07, -0.01)
Sample size	4030	4023†	3963¶	3963¶	3963¶	1487ǂ

Model specifications were as follows

Model 1. Unadjusted association of residence in intervention/control of the original trial with household agency

Model 2. Model 1 + controls for maternal education and age, household asset index, caste/ethnicity, household occupation and sex of household head

Model 3. Model 2 + controls for baseline no. deliveries and subsequent exposure to PLA groups counting only pregnancy-associated exposure

Model 4. Model 2 + controls for baseline no. deliveries and subsequent exposure to PLA groups weighting non pregnancy-associated exposure half as highly as pregnancy-associated exposure

Model 5. Model 2 + controls for baseline no. deliveries and subsequent exposure to PLA groups weighting non pregnancy-associated exposure equally highly to pregnancy-associated exposure

Model 6. Model 5 using a sample restricted to women who never delivered after the end of the original trial and never became mothers-in-law

* p<0.05

** p<0.01

*** p<0.001

^#^ Baseline number of deliveries: 14110 (Models 3–5), 4122 (Model 6).

† 7 women had missing data in at least one of the control covariates

¶ 53 additional women had missing data on baseline number of deliveries

ǂ2476 additional women excluded as they either became mothers-in-law themselves or delivered after the end of the original trial

Similarly, Table 4 shows no evidence for an association between exposure to the original intervention on any of the 4 sub-domains of agency: work outside the household, household chores, health-seeking or group participation under any model specification (Models 1–6; *p*-values all >0.05). However, we found positive, non-statistically significant relations between exposure to the original PLA group intervention and health-seeking and group participation behaviour.

**Table 4:** Impact estimates of the effect of exposure to the original Makwanpur intervention on sub-domains of the overall agency score. Effect sizes in standard deviations. 95% confidence intervals in brackets.

Model no.	1	2	3	4	5	6
*Outcome*: *Agency in work outside the household*						
Trial intervention	-0.17 (-0.53, 0.19)	-0.15 (-0.51, 0.20)	-0.16 (-0.51, 0.20)	-0.14 (-0.51, 0.23)	-0.17 (-0.60, 0.26)	-0.09 (-0.54, 0.37)
IMPEXP(0)			-0.02 (-0.08, 0.05)			
IMPEXP(0.5)				-0.02 (-0.15, 0.10)		
IMPEXP(1)					0.01 (-0.14, 0.16)	-0.03 (-0.19, 0.13)
DELIVERIES^#^			-0.01 (-0.02, 0.01)	-0.01 (-0.02, 0.01)	-0.01 (-0.02, 0.01)	0.00 (-0.03, 0.03)
*Outcome*: *Agency in household chores*						
Trial intervention	0.00 (-0.41, 0.41)	0.00 (-0.41, 0.41)	-0.01 (-0.42, 0.40)	-0.03 (-0.46, 0.39)	0.04 (-0.46, 0.53)	0.16 (-0.37, 0.69)
IMPEXP(0)			0.02 (-0.05, 0.09)			
IMPEXP(0.5)				0.03 (-0.10, 0.15)		
IMPEXP(1)					-0.03 (-0.21, 0.14)	-0.06 (-0.25, 0.13)
DELIVERIES^#^			-0.01 (-0.02, 0.01)	-0.01 (-0.02, 0.01)	-0.01 (-0.02, 0.01)	-0.03 (-0.05, 0.01)
*Outcome*: *Agency health-seeking behaviour*						
Trial intervention	0.23 (-0.29, 0.75)	0.22 (-0.29, 0.73)	0.23 (-0.28, 0.74)	0.27 (-0.25, 0.79)	0.15 (-0.47, 0.77)	0.18 (-0.44, 0.80)
IMPEXP(0)			-0.03 (-0.09, 0.02)			
IMPEXP(0.5)				-0.06 (-0.16, 0.05)		
IMPEXP(1)					0.05 (-0.18, 0.27)	0.03 (-0.20, 0.26)
DELIVERIES^#^			-0.01 (-0.02, 0.00)	-0.01 (-0.02, 0.00)	-0.01 (-0.02, 0.00)	-0.01 (-0.04, 0.01)
*Outcome*: *Agency in group participation*						
Trial intervention	0.26 (-0.07, 0.59)	0.23 (-0.09, 0.55)	0.23 (-0.07, 0.54)	0.35 (-0.01, 0.71)	0.10 (-0.26, 0.47)	0.07 (-0.29, 0.44)
IMPEXP(0)			-0.11** (-0.18, -0.04)			
IMPEXP(0.5)				-0.15* (-0.27, -0.03)		
IMPEXP(1)					0.07(-0.06, 0.20)	0.04 (-0.09, 0.17)
DELIVERIES^#^			-0.04*** (-0.05, - 0.02)	-0.04*** (-0.05, -0.02)	-0.04*** (-0.05, -0.02)	-0.05*** (-0.09, -0.02)
Sample size	4030	4023†	3963¶	3963¶	3963¶	1487ǂ

* p<0.05

** p<0.01

*** p<0.001

#baseline number of deliveries: 14110 (Models 3–5), 4122 (Model 6).

† 7 women had missing data in at least one of the control covariates

¶ 53 additional women had missing data on baseline number of deliveries

ǂ2476 additional women excluded as they either became mothers-in-law themselves or delivered after the end of the original trial

### Association between potential exposure to PLA groups post-trial and agency

Table 3 also shows the coefficients for associations between subsequent exposure to PLA groups in the same 6 model specifications with our main outcome, overall agency freedom. Potential subsequent exposure to PLA groups as modelled by IMPEXP(0) and IMPEXP(0.5), and therefore driven more by pregnancy related time after the trial, was negatively associated with overall agency (Models 3 and 4, *p*-value = 0.02 and *p*-value = 0.04 respectively), but there was no evidence for an association with IMPEXP(1) as a measure of subsequent exposure (Model 5, *p*-value = 0.64), even after restricting our sample to women who never became mothers-in-law and who never delivered again after the original trial (Model 6, *p*-value = 0.91). We found strong evidence that the baseline number of deliveries was negatively correlated with overall agency at endline (*p*-value <0.001 in Models 3–6).

Table 4 shows that we also found no evidence of an association between subsequent exposure to PLA groups (as modelled by IMPEXP(0), IMPEXP(0.5) and IMPEXP(1)) on three of the sub-domains of agency, namely: work outside the household, domestic work and health-seeking. However, we found evidence for modelling of higher levels of subsequent exposure to PLA groups being associated with reduced agency in the sub-domain of group participation, This was evident when modelling of subsequent exposure to PLA groups was driven more by pregnancy associated time in Model 3 (IMPEXP(0.5): *p*-value = 0.002) and in Model 4 (IMPEXP(1.0), *p*-value = 0.015). Whereas the association between subsequent exposure to PLA groups and sub-domain of group participation disappeared when modelling of exposure was determined more by non-pregnancy associated time (IMPEXP(1.0)) as the sign of the association became positive and statistically insignificant (Models 5 and 6, *p*-values>0.28).

Calculation of Variance Inflation Factors showed no evidence for multicollinearity. For the exposure length measure, we obtained Variance Inflation Factors of 1.02 for IMPEXP(0), 1.68 for IMPEXP(0.5) and 1.71 for IMPEXP(1), which are all well below the standard cut-off of 10. Furthermore, our findings showed only minor impacts of controlling for IMPEXP(θ) on the size of the standard errors around our main effect estimate.

**Fig 3:**
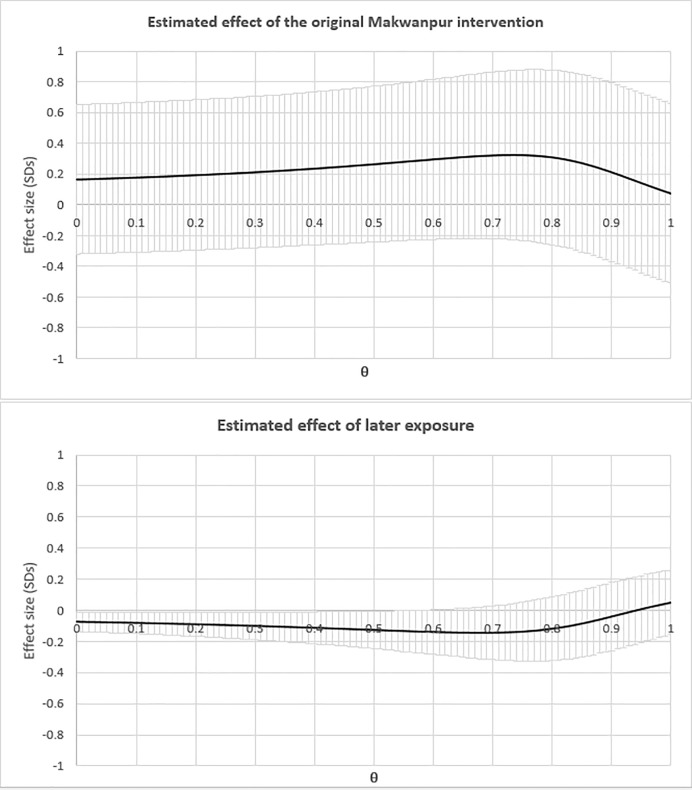
Estimates from models on the effect of the original Makwanpur intervention and subsequent exposure after varying θ in IMPEXP(θ). Effect sizes are in units of standard deviations of the overall agency measured with the RAI. Bands around point estimates of effect sizes represent 95% confidence intervals.**θ** refers to the weight given to exposure to PLA groups while non-pregnant. When **θ** we control exclusively for later exposure while pregnant. When **θ** = 1, we weight exposure to PLA groups while pregnant and non-pregnant equally.

Fig 3 displays the full range of effect sizes for the original intervention as well as subsequent exposure that was obtained by varying our estimate of later exposure to women’s groups after the end of the trial. We found that varying the weight given to women’s residence in areas running PLA groups outside of pregnancy, θ, across all possible values from 0 to 1 resulted in minimal change to estimates of the effect size of the original intervention with the vast majority of estimates between 0.2 and 0.3 SD. All the 95% CIs overlapped with zero. The effect estimates for later exposure were similarly close to zero with most estimates between -0.07 and -0.14 SD.

### Sensitivity analyses

Table 5 shows the estimated impacts on two alternative measures of agency–financial power and the ladder question. For impacts on financial power, we found neither evidence for an impact from the original intervention, nor from modelling of subsequent exposure, under any model specification (Models 1–6). For the Power Ladder question, there was a trend for residence in an intervention area during the trial being negatively associated with perceived power. However, this was statistically significant only in 3 of the 6 models, namely when additionally adjusted for socio-economic factors (Model 2), additionally adjusted for subsequent exposure to PLA groups when driven by pregnancy-related time (Mode 3) and when restricted to women who did not have any further pregnancies, nor were likely to have become a mother-in-law over that time period (Model 6).

**Table 5:** Impact estimates of the effect of exposure to the original Makwanpur intervention on alternative measures of agency. 95% confidence intervals in brackets. Effect sizes in logits for financial power. Effect sizes in steps on the Power Ladder (out of 10) for the Power Ladder question.

Model no.	1	2	3	4	5	6
*Outcome*: *Financial power*						
Trial intervention	-0.16 (-0.87, 0.56)	-0.21 (-0.92, 0.51)	-0.20 (-0.91, 0.51)	-0.09 (-0.89, 0.71)	-0.18 (-0.95, 0.59)	-0.28 (-1.33, 0.77)
IMPEXP(0)			-0.25 (-0.57, 0.08)			
IMPEXP(0.5)				-0.17 (-0.72, 0.38)		
IMPEXP(1)					-0.04 (-0.41, 0.34)	-0.07 (-0.44, 0.31)
DELIVERIES^#^			-0.04 (-0.10, 0.02)	-0.04 (-0.10, 0.02)	-0.04 (-0.10, 0.03)	-0.13 (-0.27, 0.01)
*Outcome*: *Step on the Power Ladder*						
Trial intervention	-0.27 (-0.66, 0.11)	-0.45** (-0.78, -0.11)	-0.43* (-0.78, -0.08)	-0.02 (-0.41, 0.36)	-0.39 (-0.79, 0.02)	-0.53* (-0.98, -0.08)
IMPEXP(0)			-0.33*** (-0.43, -0.23)			
IMPEXP(0.5)				-0.52*** (-0.69, -0.35)		
IMPEXP(1)					-0.06 (-0.21, 0.09)	-0.07 (-0.23, 0.09)
DELIVERIES^#^			-0.09*** (-0.12, -0.07)	-0.09*** (-0.12, -0.07)	-0.09*** (-0.11, -0.07)	-0.11*** (-0.16, -0.07)
Sample size	4030	4023†	3963¶	3963¶	3963¶	1487‡

* p<0.05

** p<0.01

*** p<0.001.

# baseline number of deliveries: 14110 (Models 3–5), 4122 (Model 6).

† 7 women had missing data in at least one of the control covariates

¶ 53 additional women had missing data on baseline number of deliveries

ǂ2476 additional women excluded as they either became mothers-in-law themselves or delivered after the end of the original trial

Similarly, models of subsequent exposure to PLA groups that were driven by further pregnancy related time (IMPEXP(0) and IMPEXP(0.5)), but not by non-pregnancy related time (IMPEXP(1)) were negatively associated with perceived power. The number of deliveries at baseline was again negatively associated with agency as measured by the Power Ladder question (*p*-value <0.001 in Models 3–6).

## Discussion

To our knowledge, this is one of the first published studies investigating the impact of PLA women’s groups in maternal and child health on women’s agency in the household with only two other studies investigating this outcome [29,30]. Long-term evaluation of participatory women’s groups is important, because “[depending] on the dimension of empowerment, the context, and the type of social, economic, or policy catalyst, women may become empowered in some aspects of their lives in a relatively short period of time (say 1–3 years) while other changes may evolve over decades” (p.80) [17]. The ability of our study to exploit a rare opportunity for long-term follow-up of women potentially exposed to participatory women’s groups is an important strength.

We found little convincing evidence for a positive relation between exposure to the original trial intervention and women’s household agency, either overall or disaggregated by subdomain. Similarly, we did not find consistent evidence of an association between subsequent exposure to PLA groups and overall household agency. There was some evidence that measures of subsequent exposure to PLA groups driven largely by subsequent time spent being pregnant were associated with reduced agency in the sub-domain of group participation. Further, in some sensitivity analyses, exposure to the original trial intervention and measures of subsequent potential exposure driven by pregnancy-related time were associated with reduced household agency in the Ladder Question. However, these later findings should be interpreted with caution as they emerged as part of sensitivity testing and robustness checking rather than due to the primary analysis.

Adjusting for trial allocation, socio-economic factors and subsequent exposure to PLA groups, the baseline number of deliveries was negatively correlated with agency freedom on average 11.5 years after enrolment into the original cRCT (irrespective of intervention allocation), but the effect size was small. The report of one additional previous pregnancy at baseline was associated with a reduction of overall agency freedom of 0.03 points (score range -12 to +12). The baseline number of deliveries was also negatively associated with perceived power as measured with the ladder question.

Our negative findings could reflect an absence of long-term impact of PLA groups on household agency with several potentially co-existing explanations. First, the original PLA intervention may not have impacted household agency at all and thus the benefits of PLA groups might be mediated through other factors–such as psychological wellbeing and/or agency outside the household. Second, PLA groups could have had a short-term impact on household agency that was not sustained after the original intervention ended. However, a cluster-randomised controlled evaluation of the impact of PLA women’s groups on resident pregnant women in Nepal found no evidence for a short-term impact on a wide range of indicators of household agency [30]. Third, the cohort at baseline may have already had levels of household agency and thus the PLA model may not have additionally improved household agency in this population. Ethnographic accounts have noted how gender norms in the Hills of Nepal are considerably less conservative than the Plains [44]. Our descriptive data suggest these women might already have had considerable agency in the family when the original intervention was trialled (Table 2). Qualitative studies of the original Makwanpur intervention also found that community readiness for change was a key contextual element affecting the success of the intervention [70].

Alternatively, our findings could be a false negative. First, it is possible that our measure of household agency–the RAI—was unable to correctly identify household agency, either because the original tool is inadequate overall or specifically in our Nepali Hill population and/or because our adaptation and translation of that tool to this setting was flawed. However, the RAI has been validated in Nepal previously [48] and sensitivity analyses with alternative measures did not reveal a positive impact on agency either. Second, large-scale secular changes in the gendered national context may have confounded intervention effects over time. However, since trial clusters were randomly assigned to intervention and control, we have no reason to believe that such national trends would have affected the local context in the Makwanpur district differently between intervention and control. It is theoretically possible that secular changes in the national context interacted with the long-term effect of the intervention by eliminating short-term gains in women’s agency in intervention compared to control after the end of the original trial. However, such an interaction would be the very definition of a lack of sustained impact and so would constitute an explanation for a lack of effect rather than a confounder.

Third, any existing differences might have become less obvious over time due to confounding by subsequent exposure to PLA groups not adequately accounted for in our models. Unlike secular trends in the national context, such exposure cannot be expected to be randomly distributed between intervention and control areas, because UCL/MIRA consciously chose to implement subsequent interventions based on their knowledge of which clusters had already previously received a PLA intervention. However, our modelling of subsequent exposure have been carefully designed, taking into account neonatal surveillance data over almost the entire post-trial period, to adjust for subsequent exposure to PLA groups arising from UCL/MIRA collaboration. In particular, our results for the main agency score did not change materially when we varied our weightings on pregnancy-related exposure, even though the proportion of pregnant women in our closed cohort decreased from 100% in the original trial period to less than a third afterwards. In our analysis of a restricted sample in which none of the women delivered again or became mothers-in-law themselves, we also found no evidence for an impact on the main agency score, even though we effectively compared a sample where 100% of women were pregnant during an intervention period with a sample where 0% of women were pregnant. The women’s groups were open to all community members, but group discussion topics revolved exclusively around perinatal health issues (see S1 Table). Even among pregnant women, the participation rate never exceeded 40% [71]. The robustness of our results to different weights on pregnancy-related exposure shows that our null result is not an artefact of how we measure subsequent exposure. Furthermore, to our knowledge there were no other governmental or non-governmental PLA group health interventions running in the area during this time period.

Finally, the finding that increased number of deliveries at baseline was associated with reduced household agency at end-line is plausible given that higher fertility has been associated with poorer socio-economic status and agency in the past [26]. This has important implications for implementation of such interventions in the future that may need to take into account baseline fertility rate in targeting beneficiaries or tailoring implementation strategies.

### Limitations

The main limitations of this study are 1) a lack of comparative and alternative measures of agency over time–both during intervention and subsequent years 2) a lack of concurrent qualitative data on perceived impact on empowerment at the community and the household level 3) low study power. Our measure of agency was limited to the household and did not address other domains–such as that related to child-care or community agency. For women, such as those in this cohort, with few demonstrable restrictions on social activities outside the household, agency in the community might be more relevant. Measures of social support [72], social capital [73] or community mobilisation [74,75] could be gainfully used to assess the extent to which PLA women’s groups empower women at a community level as opposed to a household level. In particular, the ability of PLA women’s groups to lower transaction costs to women’s collective action and enhance their capacity to manage communal resources is arguably a key social outcome of the participatory approach [76].

Additionally, subjective measures are frequently criticised for being subject to cognitive and social biases, easily influenced by the framing of questions, and unable to reveal causal factors that are not accessible to introspection [77]. Over the 11.5 years from baseline to end-line, life aspirations and preferences may have changed and could lead to women feeling disempowered due to higher aspirations rather than lower ‘objective conditions’. The Power Ladder question likely measures relative power, since it requires respondents to anchor the 10-point scale using the perceived agency of other women ‘in their community’. Higher levels of agency among community women in general may lead to feelings of low agency when women compare themselves with each other rather than their past selves. However, we should note that more ‘objective’ measures of social constructs based on observed behaviour in the field or in the lab often imply equally, if not more, challenging interpretive difficulties due to the need to decode the meaning of ambiguous human behaviour. We recommend using both behavioural and subjective measures of empowerment in future research.

Finally, the precision of our effect estimates is limited. This was a post-hoc study and the original study sample size calculations, in particular determining the number of clusters, was not powered with this outcome in mind. A post-hoc power analysis based on an observed ICC of 0.36 for overall agency, 24 clusters in total and 256 individuals per cluster suggested we had 80% power to detect an absolute 0.23 standard deviation difference in mean overall household agency between intervention and control at 5% significance level [78]. As stated previously, the mean overall agency freedom score for all women, irrespective of trial arm, was 9.6 with astandard deviation of 2.2.

## Conclusion

We investigated the long-term impacts of a perinatal PLA women’s group intervention on women’s household agency approximately 11.5 years after individuals’ original exposure to the intervention. There was no robust evidence to support a long-term impact of the original intervention on women’s agency in the family according to a wide range of model specifications. Without in-depth qualitative evidence and/or interim measures of household agency and other measures of agency, it is difficult to draw firm conclusions about the reasons for our lack of observed impact. Our study does, however, highlight important methodological considerations that should be taken into account when addressing the question of unpicking potential pathways to impact–at the individual, family and community level of community-based mobilisation interventions to improve maternal and child health. Future work should collect qualitative and quantitative process and implementation data over time in order to better understand the mechanisms through which women’s groups and similar participatory and community based interventions improve health outcomes.

## Supporting information

S1 Table Activity schedule for all participatory women’s group meetings 2001–2014. (DOCX)

S2 Table Statements for the main survey module. (DOCX)

## References

[1] Prost A, Colbourn T, Seward N, Azad K, Coomarasamy A, Copas A, et al Women's groups practising participatory learning and action to improve maternal and newborn health in low-resource settings: a systematic review and meta-analysis. The Lancet 2013;381(9879):1736–46. 10.1016/S0140-6736(13)60685-6 PMC3797417 23683640

[2] Freire P. Pedagogy of Oppressed. New York: Herder and Herder; 1972.

[3] World Health Organization. WHO recommendation on community mobilization through facilitated participatory learning and action cycles with women's groups for maternal and newborn health. Geneva: World Health Organization; 2014. 25165805

[4] Victora CG, Barros FC. Participatory women's groups: ready for prime time? The Lancet 381(9879):1693–4. 10.1016/S0140-6736(13)61029-6 23683618

[5] Victora CG. Commentary: Participatory interventions reduce maternal and child mortality among the poorest, but how do they work? International journal of epidemiology 2013;42(2):503–5. doi: 10.1093/ije/dyt044 23569190 10.1093/ije/dyt044

[6] Morrison J, Thapa R, Sen A, Neupane R, Borghi J, Tumbahangphe KM, et al Utilization and management of maternal and child health funds in rural Nepal. Community Development Journal 2010;45(1):75–89. doi: 10.1093/cdj/bsn029 28824196 10.1093/cdj/bsn029 PMC5562271

[7] Morrison J, Tamang S, Mesko N, Osrin D, Shrestha B, Manandhar M, et al Women's health groups to improve perinatal care in rural Nepal. BMC pregnancy and childbirth 2005;5(1):6 doi: 10.1186/1471-2393-5-6 15771772 10.1186/1471-2393-5-6 PMC1079874

[8] Rosato M. How does community mobilisation through MaiMwana women’s groups work? Addressing the social determinants of mother and child health in rural Malawi [Thesis (Ph.D.)]. London: University College London; 2012.

[9] Rath S, Nair N, Tripathy PK, Barnett S, Rath S, Mahapatra R, et al Explaining the impact of a women's group led community mobilisation intervention on maternal and newborn health outcomes: the Ekjut trial process evaluation. BMC International health and human rights 2010;10(1):25. 20969787 10.1186/1472-698X-10-25 PMC2987759

[10] Rosato M, Malamba F, Kunyenge B, Phiri T, Mwansambo C, Kazembe P, et al Strategies developed and implemented by women's groups to improve mother and infant health and reduce mortality in rural Malawi. International health 2012;4(3):176–84. doi: 10.1016/j.inhe.2012.03.007 24029397 10.1016/j.inhe.2012.03.007

[11] Morrison J, Thapa R, Hartley S, Osrin D, Manandhar M, Tumbahangphe K, et al Understanding how women's groups improve maternal and newborn health in Makwanpur, Nepal: a qualitative study. International health 2010;2(1):25–35. doi: 10.1016/j.inhe.2009.11.004 24037047 10.1016/j.inhe.2009.11.004 PMC5104268

[12] Every Women Every Child. Global strategy for women's children's and adolescents' health 2016–2030. United Nations Sustainable Development Goals 2017 July 9 [cited 2017 Jul 15];Available from: URL: http://globalstrategy.everywomaneverychild.org/pdf/EWEC_globalstrategyreport_200915_FINAL_WEB.pdf

[13] UN General Assembly. Transforming our world: the 2030 Agenda for Sustainable Development. 2015.

[14] Drydyk J. Empowerment, agency, and power. Journal of Global Ethics 2013;9(3):249–62.

[15] Trommlerová SK, Klasen S, Lessmann O. Determinants of Empowerment in a Capability-Based Poverty Approach: Evidence from The Gambia. World Development 2015;66:1–15.

[16] Kabeer N. Resources, agency, achievements: Reflections on the measurement of women's empowerment. Development and Change 1999;30(3):435–64.

[17] Malhotra A, Schuler SR. Women’s empowerment as a variable in international development In: Narayan D, editor. Measuring empowerment: Cross-disciplinary perspectives. Washington DC: World Bank; 2005 p. 71–88.

[18] Rowlands J. Questioning empowerment: Working with Women in Honduras. Oxford: Oxfam; 1997.

[19] Ibrahim S, Alkire S. Agency and Empowerment: A Proposal for Internationally Comparable Indicators. Oxford development studies 2007;35(4):379–403.

[20] Alsop R, Bertelsen M, Holland J. Empowerment in practice: From analysis to implementation. Washington, DC: World Bank; 2006.

[21] Yount KM, VanderEnde KE, Dodell S, Cheong YF. Measurement of women's agency in Egypt: A National Validation Study. Social indicators research 2016;128(3):1171–92. doi: 10.1007/s11205-015-1074-7 27597801 10.1007/s11205-015-1074-7 PMC5010232

[22] Sen A. Well-being, agency and freedom: the Dewey lectures 1984. The Journal of Philosophy 1985;82(4):169–221.

[23] Putnam H. The collapse of the fact/value dichotomy and other essays. Cambridge, MA and London, England: Harvard University Press; 2002.

[24] Jack DC, Pokharel B, Subba U. ‘I don’t express my feelings to anyone’: How self-silencing relates to gender and depression in Nepal. Silencing the self across cultures: Depression and gender in the social world 2010;147–75.

[25] Mandelbaum DG. Women's seclusion and men's honor: Sex roles in North India, Bangladesh, and Pakistan. Tucson and London: University of Arizona Press; 1993.

[26] Upadhyay UD, Gipson JD, Withers M, Lewis S, Ciaraldi EJ, Fraser A, et al Women's empowerment and fertility: A review of the literature. Social Science & Medicine 2014;115:111–20. 24955875 10.1016/j.socscimed.2014.06.014 PMC4096045

[27] Cunningham K, Ruel M, Ferguson E, Uauy R. Women's empowerment and child nutritional status in South Asia: a synthesis of the literature. Maternal & child nutrition 2014;11(1):1–19. 24850533 10.1111/mcn.12125 PMC6860351

[28] Pratley P. Associations between quantitative measures of women's empowerment and access to care and health status for mothers and their children: A systematic review of evidence from the developing world. Social Science & Medicine 2016;169:119–31. 27716549 10.1016/j.socscimed.2016.08.001

[29] Harris-Fry HA, Azad K, Younes L, Kuddus A, Shaha S, Nahar T, et al Formative evaluation of a participatory women9s group intervention to improve reproductive and women9s health outcomes in rural Bangladesh: a controlled before and after study. J Epidemiol Community Health 2016;jech-2015. 10.1136/jech-2015-205855 PMC4941186 26739272

[30] Gram L, Morrison J, Saville N, Shrestha BP, Yadav SS, Manandhar D.S., et al The Impact of Participatory Learning and Action Women's Groups Alone or Combined with Cash and Food Transfers on Women's Agency in Rural Nepal. Journal of Development Studies. In press 2018. 10.1080/00220388.2018.1448069 PMC6540743 31218298

[31] Manandhar DS, Osrin D, Shrestha BP, Mesko N, Morrison J, Tumbahangphe KM, et al Effect of a participatory intervention with women's groups on birth outcomes in Nepal: cluster-randomised controlled trial. The Lancet 2004;364(9438):970–9. 10.1016/S0140-6736(04)17021-9 15364188

[32] World Bank. Data—Nepal. World Bank. 2018.

[33] World Bank. Personal remittances, received (% of GDP). 2018. 27-1-2018.

[34] World Bank. Employment in agriculture (% of total employment) (modeled ILO estimate). 2018. 27-1-2018.

[35] Ministry of Health and Population (MOHP) [Nepal], New ERA, Macro International Inc. Nepal Demographic and Health Survey 2006. Kathmandu, Nepal: Ministry of Health and Population (MOHP) [Nepal]; New ERA; Macro International Inc.; 2007.

[36] Ministry of Health and Population (MOHP) [Nepal], New ERA, Macro International Inc. Nepal Demographic and Health Survey 2016. Kathmandu, Nepal: Ministry of Health and Population (MOHP) [Nepal]; New ERA; Macro International Inc.; 2017.

[37] Tamang S. the politics of conflict and difference or the difference of conflict in politics: the women's movement in Nepal. Feminist Review 2009;91(1):61–80.

[38] Sharma M, Prasain D. Gender Dimensions of the People's War In: Hutt M, editor. Himalayan people's war: Nepal's Maoist rebellion. Bloomgton and Indianapolis: Indiana University Press; 2004 p. 152–65.

[39] Pant B, Standing K. Citizenship rights and women's roles in development in post-conflict Nepal. Gender & Development 2011;19(3):409–21.

[40] World Bank. Gender Data Portal—Nepal. 2018. 27-1-2018.

[41] World Bank. Labor force participation rate, female (% of female population ages 15+) (modeled ILO estimate). 2018. 27-1-2018.

[42] Ministry of Health and Population (MOHP) [Nepal], New ERA, Macro International Inc. Nepal Demographic and Health Survey 2001. Kathmandu, Nepal: Ministry of Health and Population (MOHP) [Nepal]; New ERA; Macro International Inc.; 2002.

[43] Statistics CB. National Population and Housing Census. Government of Nepal; 2012.

[44] Acharya M, Bennett L. Women and the Subsistence Sector. Economic Participation and Household Decision-making in Nepal. Washington, D.C.: World Bank; 1983. World Bank Staff Working Papers. Report No.: 526.

[45] Morrison J, Tumbahangphe KM, Budhathoki B, Neupane R, Sen A, Dahal K, et al Community mobilisation and health management committee strengthening to increase birth attendance by trained health workers in rural Makwanpur, Nepal: study protocol for a cluster randomised controlled trial. Trials 2011;12(1):1. 21595902 10.1186/1745-6215-12-128 PMC3121607

[46] CommCare main web site. https://wwwcommcarehqorg/home/2016

[47] World Health Organization. Assisting community health workers in India: Dimagi's CommCare 2013.

[48] Gram L, Morrison J, Sharma N, Shrestha BP, Manandhar, Costello A, et al Validating an Agency-Based Tool for Measuring Women's Empowerment in a Complex Public Health Trial in Rural Nepal. Journal of Human Development and Capabilities 2016;1–29. 10.1080/19452829.2016.1251403 PMC5327873 28303173

[49] Alkire S. Measuring the Freedom Aspects of Capabilities. Cambridge, MA: Global Equity Initiative, Harvard University; 2005.

[50] Alkire S. Subjective quantitative studies of human agency. Social indicators research 2005;74(1):217–60.

[51] Deci EL, Ryan RM. The" what" and" why" of goal pursuits: Human needs and the self-determination of behavior. Psychological inquiry 2000;11(4):227–68.

[52] Vaz A, Alkire S, Quisumbing A, Sraboni E. Measuring Autonomy: Evidence from Bangladesh. Oxford Poverty and Human development Initiative 2013 August 31 [cited 2016 Oct 11];Available from: URL: https://assets.publishing.service.gov.uk/media/57a08a4640f0b649740004fc/60954_Measuring_Autonomy_1.pdf

[53] Vaz A, Pratley P, Alkire S. Measuring Women's Autonomy in Chad Using the Relative Autonomy Index. Feminist economics 2016;22(1):264–94.

[54] Sarkar K, Dasgupta A, Sinha M, Shahbabu B. Effects of health empowerment intervention on resilience of adolescents in a tribal area: A study using the Solomon four-groups design. Social Science & Medicine 2017;190:265–74. 28625414 10.1016/j.socscimed.2017.05.044

[55] Patrick H, Williams GC. Self-determination theory: its application to health behavior and complementarity with motivational interviewing. International Journal of behavioral nutrition and physical Activity 2012;9(1):18. 22385676 10.1186/1479-5868-9-18 PMC3323356

[56] Alkire S, Meinzen-Dick R, Peterman A, Quisumbing A, Seymour G, Vaz A. The women’s empowerment in agriculture index. World Development 2013;52:71–91.

[57] Sraboni E, Malapit HJ, Quisumbing AR, Ahmed AU. Women’s Empowerment in Agriculture: What Role for Food Security in Bangladesh? World Development 2014;61:11–52.

[58] Malapit HJ, Quisumbing AR. What dimensions of women's empowerment in agriculture matter for nutrition in Ghana? Food Policy 2015;52:54–63.

[59] Malapit HJ, Kadiyala S, Quisumbing AR, Cunningham K, Tyagi P. Women's Empowerment in Agriculture, Production Diversity, and Nutrition: Evidence from Nepal IFPRI Discussion Paper. Washington, D.C.: International Food Policy Research Institute, Poverty Health and Nutrition Division; 2013.

[60] Malapit HJ, Kadiyala S, Quisumbing AR, Cunningham K, Tyagi P. Women's empowerment mitigates the negative effects of low production diversity on maternal and child nutrition in Nepal. The Journal of Development Studies 2015;51(8):1097–123.

[61] Bastagli F, Hagen-Zanker J, Harman L, Barca V, Sturge G, Schmidt T. Cash transfers: what does the evidence say? A rigorous review of programme impact and the role of design and implementation features. London: Overseas Development Institute; 2016.

[62] Brody C, De Hoop T, Vojtkova M, Warnock R, Dunbar M, Murthy P, et al. Economic Self-Help Group Programs for Improving Women's Empowerment: A Systematic Review. The Campbell Collaboration; 2015. Campbell Systematic Reviews 2015. Report No.: 19.

[63] Gram L. Women's Empowerment in a Complex Public Health Intervention in Rural Nepal [Thesis (Ph.D.)]. London: UCL; 2018.

[64] Alkire S, Meinzen-Dick R, Peterman A, Quisumbing AR, Seymour G, Vaz A. The Women's Empowerment in Agriculture Index. Washington, D.C.: International Food Policy Research Institute; 2012.

[65] Lokshin M, Ravallion M. Rich and powerful?: Subjective power and welfare in Russia. Journal of economic behavior & organization 2005;56(2):141–72.

[66] Provan KG. Recognizing, measuring, and interpreting the potential/enacted power distinction in organizational research. Academy of Management Review 1980;5(4):549–59.

[67] Iyengar SS, Lepper MR. When choice is demotivating: Can one desire too much of a good thing? Journal of personality and social psychology 2000;79(6):995 11138768 10.1037//0022-3514.79.6.995

[68] O'brien RM. A caution regarding rules of thumb for variance inflation factors. Quality & Quantity 2007;41(5):673–90.

[69] StataCorp LP. Stata 13. College Station: StataCorp LP 2014.

[70] Morrison J. Understanding the effect of a participatory intervention with women's groups to improve maternal and neonatal health in rural Nepal [Thesis (Ph.D.)]. London: UCL (University College London); 2009.

[71] Houweling TA, Morrison J, Alcock G, Azad K, Das S, Hossen M, et al Reaching the poor with health interventions: programme-incidence analysis of seven randomised trials of women's groups to reduce newborn mortality in Asia and Africa. Journal of epidemiology and community health 2015;jech-2014. 10.1136/jech-2014-204685 PMC4717375 26246540

[72] Gottlieb BH, Bergen AE. Social support concepts and measures. Journal of psychosomatic research 2010;69(5):511–20. doi: 10.1016/j.jpsychores.2009.10.001 20955871 10.1016/j.jpsychores.2009.10.001

[73] Pronyk PM, Harpham T, Busza J, Phetla G, Morison LA, Hargreaves JR, et al Can social capital be intentionally generated? A randomized trial from rural South Africa. Social Science & Medicine 2008;67(10):1559–70. 18771833 10.1016/j.socscimed.2008.07.022

[74] Lippman SA, Neilands TB, Leslie HH, Maman S, MacPhail C, Twine R, et al Development, validation, and performance of a scale to measure community mobilization. Social Science & Medicine 2016;157:127–37. 27085071 10.1016/j.socscimed.2016.04.002 PMC4853196

[75] Altman L, Kuhlmann AKS, Galavotti C. Understanding the black box: A systematic review of the measurement of the community mobilization process in evaluations of interventions targeting sexual, reproductive, and maternal health. Evaluation and program planning 2015;49(1):86–97. 25615599 10.1016/j.evalprogplan.2014.11.010

[76] Ostrom E. Collective action and the evolution of social norms. Journal of Natural Resources Policy Research 2014;6(4):235–52.

[77] Bertrand M, Mullainathan S. Do people mean what they say? Implications for subjective survey data. American Economic Review 2001;91(2):67–72.

[78] Donner A, Klar N. Design and analysis of cluster randomization trials in health research. London: Arnold; 2000.

