# Supplementary material for: The long-term impact of community mobilisation through participatory women's groups on women's agency in the household: A follow-up study to the Makwanpur trial
Source: PLoS One. 2018 May 14;13(5):e0197426. doi: 10.1371/journal.pone.0197426 (PMC5951552; doi:10.1371/journal.pone.0197426)
Supplement: S1 Table — (DOCX) [file pone.0197426.s001.docx]

| *Dates* | *Nov 2001 - Jun 2005 for Regions 1 and 2 Jul 2005 - Dec 2008 for Regions 3 and 4 Jul 2006 - Dec 2008 for Regions 5 and 6* |
| --- | --- |
| **Meeting** | **Topic** |
| 1 | To introduce the study to the group |
| 2 | To discuss why mothers and newborn infants die and how the intervention will work in the community |
| 3 | To ascertain how women understand maternal and neonatal problems |
| 4 | To find out about maternal and neonatal problems in the community |
| 5 | To understand the frequency of maternal and neonatal problems and to identify strategies to obtain information in the community |
| 6 | To share information from other women in the community and to prioritise three important maternal and neonatal health problems |
| 7 | To discuss possible strategies for addressing the priority problems |
| 8 | To discuss involvement of other community members in developing strategies |
| 9 | To discuss preparation for a meeting of community members |
| 10 | To hold a meeting involving other community members to discuss the activities of the women's groups, the priority problems identified by the groups, and possible strategies, and reach consensus |
|  |  |
| *Dates* | *Jul 2005 - Dec 2007 for Regions 1 and 2* |
| **Meeting** | **Topic** |
| 1 | Introduce the new topic (caring for childhood illness) and how we plan to work |
| 2 | Plan how to inform the community of the new focus on child health, and who to invite Plan how to continue existing work with newborn and maternal health |
| 3 | Introduce new members to the work of the group and the topic of child health and discuss the reasons why children die |
| 4 | To discuss illnesses that affect children, to explore reasons why they have this illness and to explore what action is taken when children have these illnesses |
| 5 | To discuss what criteria will be used in prioritising problems and to discuss how to collect data useful for prioritisation |
| 6 | To share information collected and to prioritise problems to address through strategies |
| 7 | To identify local resources available, and discuss ways to overcome prioritised problems To discuss if health personnel should be invited to the meeting |
| 8 | To learn about medical causes and medical treatments for prioritised problems To discuss strategies with health personnel (if they are available) |
| 9 | To discuss how illness are obtained and passed using different social games |
| 10 | Discuss the feasibility of strategies and how to disseminate strategies discussed |
| 11 | To share information with the community and plan how to implement the strategies |
| 12 | To discuss how the group will know if the strategies have been effective or not To plan how these strategies will be evaluated |
|  |  |
| *Dates* | *Jan 2008 - Jun 2008 for Regions 1 and 2* |
| **Meeting** | **Topic** |
| 1 | To discuss family decision-making in the ward and to discuss men's role in maternal and newborn care |
| 2 | To discuss barriers preventing supportive behaviour of men and to plan how to work with men to overcome these barriers |
| 3 | To plan how to work with men, talk to men, invite men to participate and evaluate the effectiveness of the chosen strategy |
| 4 | The women's group and local men meet to discuss their agenda and plan how to work together in the future |
|  |  |
| *Dates* | *Oct 2010 - Sep 2012 for Regions 2, 4 and 6* |
| **Meeting** | **Topic** |
| 1 | Introducing MIRA, working in groups and the importance of institutional delivery |
| 2 | Discussing community barriers to institutional delivery and how they can be overcome in general |
| 3 | Sharing personal experiences and information about community barriers to institutional delivery |
| 4 | Prioritising barriers to address through participatory voting and discussing strategies going forward |
| 5 | Preparing a community meeting to present barriers to institutional delivery and ways to address them |
| 6 | Holding a community meeting about addressing barriers to institutional delivery |
| 7 | Discussing the implementation of strategies and plan cluster-level interaction with other groups |
| 8 | Carrying out a cluster-level interaction with other groups in the community |
| 9 | Carrying out an internal evaluation of the work of the group |
| 10 | Planning an evaluation of the strategies the group has carried out |
| 11 | Presenting the results from the evaluation to the community |
| 12 | Planning how to improve existing strategies |
