# Supplementary material for: The long-term impact of community mobilisation through participatory women's groups on women's agency in the household: A follow-up study to the Makwanpur trial
Source: PLoS One. 2018 May 14;13(5):e0197426. doi: 10.1371/journal.pone.0197426 (PMC5951552; doi:10.1371/journal.pone.0197426)
Supplement: S2 Table — (DOCX) [file pone.0197426.s002.docx]

|  |  | **Scoring** |  |
| --- | --- | --- | --- |
| *Questions* | *Internal motivation* | *External motivation* | *Agency Freedom* |
| **Domain: Work outside the home** |  |  |  |
| What type of work are you normally involved in?  (Work inside the home, work outside the home or both) | | | |
| [If she performs work outside] What type of work outside the home are you normally involved in? (Daily paid labour, regular job, farming, etc.) | | | |
| [If the respondent performs work outside] Please indicate whether you agree or disagree with the following statements by saying "yes" or "no": | | | |
| a) You do this work because you will get in trouble if you don’t. | 0 | +3/4 | -3/4 |
| b) You do this work because you want to. | +1 | 0 | +1 |
| c) You do this work because that is what your family members tell you to do. | 0 | +3/4 | -3/4 |
| d) You do this work because you personally think it is the right thing to do, whether or not your family members agree. | +1 | 0 | +1 |
| e) You do this work because you like it. | +1 | 0 | +1 |
| f) You do this work so that your family members won’t get angry with you. | 0 | +3/4 | -3/4 |
| g) You do this work because you want your family members to like you. | 0 | +3/4 | -3/4 |
| [If the respondent does not perform work outside] Please indicate whether you agree or disagree with the following statements by saying "yes" or "no": | | | |
| a) You do not work outside the home because you will get in trouble if you do. | 0 | +3/4 | -3/4 |
| b) You do not work outside the home because you do not want to. | +1 | 0 | +1 |
| c) You do not work outside the home because your family members tell you not to do so. | 0 | +3/4 | -3/4 |
| d) You do not work outside the home because you personally think it is the right thing to do, whether or not your family members agree. | +1 | 0 | +1 |
| e) You do not work outside the home because you don’t like it. | +1 | 0 | +1 |
| f) You do not work outside the home because your family members might get angry with you if you do. | 0 | +3/4 | -3/4 |
| g) You do not work outside the home because you want your family members to like you. | 0 | +3/4 | -3/4 |

|  |  | **Scoring** |  |
| --- | --- | --- | --- |
| *Questions* | *Internal motivation* | *External motivation* | *Agency Freedom* |
| **Domain: Household chores** |  |  |  |
| In your household, who does each of these things most of the time: *Preparing and cooking the main meal, shopping and bringing food for the household, cleaning the home, cleaning dishes and doing laundry, doing household repairs, looking after household money and paying bills, spending time with children and caring for them when they are ill, looking after elderly or ill people, teaching children good behaviour* | | | |
| [Referring to the activities that the respondent is engaged in] Please indicate whether you agree or disagree with the following statements by saying "yes" or "no": | | | |
| a) You do these activities because you will get in trouble if you don’t. | 0 | +3/4 | -3/4 |
| b) You do these activities because you want to. | +1 | 0 | +1 |
| c) You do these activities because that is what your family members tell you to do. | 0 | +3/4 | -3/4 |
| d) You do these activities because you personally think it is the right thing to do, whether or not your family members agree. | +1 | 0 | +1 |
| e) You do these activities because you like doing them. | +1 | 0 | +1 |
| f) You do these activities so that your family members won’t get angry with you. | 0 | +3/4 | -3/4 |
| g) You do these activities because you want your family members to like you. | 0 | +3/4 | -3/4 |
| **Domain: Health-seeking behaviour** |  |  |  |
| Where would you go if you had simple health problems, such as a severe headache or a painful tooth ache? (*A public sector health institution, private medical centre, pharmacy/medical shop, other, would not do anything*) | | | |
| [If the respondent answers 'would not do anything'] Please indicate whether you agree or disagree with the following statements by saying "yes" or "no": | | | |
| a) You don’t do anything because you will get in trouble if you do. | 0 | +3/4 | -3/4 |
| b) You don’t do anything because you don’t want to do anything. | +1 | 0 | +1 |
| c) You don’t do anything because that is what your family members tell you to do. | 0 | +3/4 | -3/4 |
| d) You don’t do anything because you personally think it is the right thing to do whether or not your family members agree. | +1 | 0 | +1 |
| e) You don’t do anything because you don’t like doing anything. | +1 | 0 | +1 |
| f) You don’t do anything so that your family members won’t get angry with you. | 0 | +3/4 | -3/4 |
| g) You don’t do anything because you want your family members to like you. | 0 | +3/4 | -3/4 |

|  |  | **Scoring** |  |
| --- | --- | --- | --- |
| *Questions* | *Internal motivation* | *External motivation* | *Agency Freedom* |
| [If the respondent does not answer 'would not do anything'] Please indicate whether you agree or disagree with the following statements by saying "yes" or "no": | | | |
| a) You seek health care the way you do because you will get in trouble if you don’t. | 0 | +3/4 | -3/4 |
| b) You seek health care the way you do because you want to. | +1 | 0 | +1 |
| c) You seek health care the way you do because that is what your family members tell you to do. | 0 | +3/4 | -3/4 |
| d) You seek heath care the way you do because you personally think it is the right thing to do, whether or not your family members agree. | +1 | 0 | +1 |
| e) You seek health care the way you do because you like doing it more than other alternatives. | +1 | 0 | +1 |
| f) You seek health care the way you do so that your family members won’t get angry with you | 0 | +3/4 | -3/4 |
| g) You seek health care the way you do because you want your family members to like you. | 0 | +3/4 | -3/4 |
| **Domain: Group participation** |  |  |  |
| Do you participate in any group, organisation, network, association, etc.? | | | |
| [If the respondent participates in groups] What kind of organisations do you belong to? (*relates to main economic activity, deals with finance, credit or savings, deals with health issues, deals with education issues, political groups or associations, religious groups or associations, ethnic groups or associations, other*) | | | |
| [If the respondent participates in groups] Which is the most important group or association that you belong to? | | | |
| [If the respondent participates in groups] Please indicate whether you agree or disagree with the following statements by saying "yes" or "no": | | | |
| a) You participate because you will get in trouble if you don’t. | 0 | +3/4 | -3/4 |
| b) You participate because you want to participate. | +1 | 0 | +1 |
| c) You participate because that is what your family members tell you to do. | 0 | +3/4 | -3/4 |
| d) You participate because you personally think it is the right thing to do, whether or not your family members agree. | +1 | 0 | +1 |
| e) You participate because you like it. | +1 | 0 | +1 |
| f) You participate so that your family members won’t get angry with you. | 0 | +3/4 | -3/4 |
| g) You participate because you want your family members to like you. | 0 | +3/4 | -3/4 |

|  |  | **Scoring** |  |
| --- | --- | --- | --- |
| *Questions* | *Internal motivation* | *External motivation* | *Agency Freedom* |
| [If the respondent does not participate in groups] Please indicate whether you agree or disagree with the following statements by saying "yes" or "no": | | | |
| a) You don’t participate because you will get in trouble if you do. | 0 | +3/4 | -3/4 |
| b) You don’t participate because you don’t want to. | +1 | 0 | +1 |
| c) You don’t participate because your family members tell you not to. | 0 | +3/4 | -3/4 |
| d) You don’t participate because you personally think it is the right thing to do, whether or not your family members agree. | +1 | 0 | +1 |
| e) You don’t participate because you don’t like participating. | +1 | 0 | +1 |
| f) You don’t participate so that your family members won’t get angry with you. | 0 | +3/4 | -3/4 |
| g) You don’t participate because you want your family members to like you. | 0 | +3/4 | -3/4 |
